# Steroid-refractory chronic graft-versus-host disease: treatment options and patient management

**DOI:** 10.1038/s41409-021-01389-5

**Published:** 2021-07-03

**Authors:** Daniel Wolff, Giancarlo Fatobene, Vanderson Rocha, Nicolaus Kröger, Mary E. Flowers

**Affiliations:** 1 Department of Internal Medicine III, University Hospital Regensburg, Regensburg, Germany; 2 Hospital das Clinicas da Faculdade de Medicina da Universidade de São Paulo (HC-FMUSP), São Paulo, Brazil; 3 Vila Nova Star Hospital and IDOR, Rede D’Or, São Paulo, Brazil; 4 Department of Stem Cell Transplantation, University Medical Center Hamburg, Hamburg, Germany; 5 Clinical Research Division, Fred Hutchinson Cancer Research Center and Department of Medicine, University of Washington, Seattle, Seattle, WA USA

**Keywords:** Graft-versus-host disease, Drug therapy

## Abstract

Chronic graft-versus-host disease (cGVHD) is one of the major causes of late mortality after allogenic hematopoietic stem cell transplantation. Moderate-to-severe cGVHD is associated with poor health-related quality of life and substantial disease burden. While corticosteroids with or without calcineurin inhibitors comprise the first-line treatment option, the prognosis for patients with steroid-refractory cGVHD (SR-cGVHD) remains poor. The mechanisms underlying steroid resistance are unclear, and there are no standard second-line treatment guidelines for patients with SR-cGVHD. In this review, we provide an overview on current treatment options of cGVHD and use a series of theoretical case studies to elucidate the rationale of choices of second- and third-line treatment options for patients with SR-cGVHD based on individual patient profiles.

## Introduction

The use of allogenic hematopoietic stem cell transplant (allo-HSCT) for treatment of malignant and non-malignant conditions continues to increase annually due to significant improvement in early mortality [1], but moderate or severe chronic graft-versus-host disease (cGVHD) remains a major limitation for broadening the allo-HSCT clinical application [2] with patients developing steroid-refractory cGVHD (SR-cGVHD) having a significantly increased morbidity and mortality [3]. SR-cGVHD has been defined as cGVHD progression while on prednisone at ≥1 mg/kg/day for 1–2 weeks, or stable cGVHD while on ≥0.5 mg/kg/day for 1–2 months, and additional patients remain steroid-dependent with repeated symptom flares during taper of corticosteroids below 0.25 mg/kg/day [4].

The National Institutes of Health (NIH) consensus criteria define acute GVHD (aGVHD) and cGVHD based on a combination of clinical features and time of onset [5]. Approximately 30–70% of allo-HSCT recipients surviving at least 100 days post-transplant develop cGVHD [6]. Increasing rates have been reported in recent decades [7] due to several factors, including increased use of granulocyte colony-stimulating factor mobilized blood stem cells as the graft source, increased patient´s age, and use of unrelated as well as HLA-mismatch donors with the previous aGVHD, further increasing risk [8, 9].

**Fig. 1:**
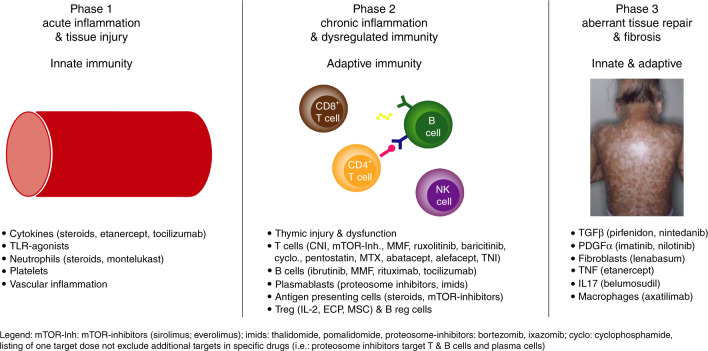
Biologic phases of c-GVHD with agents added targeting specific pathways (modified version derived from Cooke 2017 [24]). *CNI* calcineurin inhibitors, *cyclo*. cyclophosphamide, *ECP* extracorporeal photopheresis, *IL-2* interleukin-2, *IL17* interleukin 17, *imids* immunomodulatory imide drugs, *MMF* mycophenolate mofetil, *MSC* mesenchymal stromal cells, *mTOR-Inh*. mechanistic target of rapamycin inhibitors, *MTX* methotrexate, *PDGFα* platelet-derived growth factor receptor alpha, *TGFβ* transforming growth factor β, *TLR* Toll-like receptor, *TNFα* tumor necrosis factor alpha, *TNI* total nodal irradiation. mTOR-Inh.: sirolimus, everolimus; imids: thalidomide, pomalidomide; proteosome inhibitors: bortezomib, ixazomib. It should be noted that listing of one target does not exclude additional targets in specific drugs (i.e. proteosome inhibitors target T cells, B cells, and plasma cells).

Unlike in aGVHD, the underlying pathogenesis of cGVHD is not well understood, but is thought to be complex and multifactorial, with involvement of T and B cells as well as innate responses, including the transition from inflammation to fibrosis involving fibroblasts and macrophages [10–12]. Decreased regulatory T cells (CD4 + CD25 + ) have been observed in cGVHD, which may increase the proliferation of type 1 T cells [13]. Increased levels of transforming growth factor-β are observed in patients with cGVHD compared with healthy controls, although its role in disease pathogenesis has not been established [14, 15]. Additionally, auto-antibody production by host-reactive B cells and plasmablasts, fibrotic changes following type 2 donor responses, and thymic damage impairing immune-reconstitution contribute to the pathogenesis [10, 15]. Figure 1 contains a summary of the pathophysiology of cGVHD including treatment options targeting specific pathways.

There is also an emerging understanding of the role of the gut microbiome on cGVHD presentation [16]. Loss of flora diversity following allo-HSCT has been associated with the development of aGVHD and cGVHD, as well as increased mortality risk [17]. However, the specific role of these intestinal changes in cGVHD has not been studied as thoroughly as in aGVHD.

Signs and symptoms of cGVHD can be stratified as diagnostic, distinctive, and those in common with aGVHD [5], but patients can also show manifestations of other immune-mediated disease such as Hashimoto’s thyroiditis or glomerulonephritis (“other/associated” manifestations) [4]. Correct diagnosis is crucial for the treatment of cGVHD. Of note, a significant proportion of patients lack diagnostic signs of cGVHD and several case series showed that a relevant subgroup of patients being treated for cGVHD in the absence of histopathological confirmation had other diseases, not GVHD [18, 19]. Therefore, patients not responding to treatment for suspected cGVHD and lacking diagnostic symptoms, should be re-evaluated by histopathology [20, 21].

Clinical features may affect multiple organs or body areas, with varying presentation depending on the site involved [6, 10]. The skin is most commonly affected, observed in up to 75% of cGVHD cases; symptoms and signs include poikiloderma, lichen planus-like eruptions, sclerotic features, and depigmentation [5]. The presence and severity of organ-specific signs and symptoms (NIH score 0 to 3) contribute to the NIH global severity of cGVHD (mild, moderate, or severe) [5]. While overall severity has a significant impact on morbidity and mortality, additional risk factors for increased mortality consistently reported include direct progression from aGVHD to cGVHD, and platelets <100/nL at time of diagnosis [22]. Moreover, certain organ involvement such as the lung and gastrointestinal tract, and hyperbilirubinemia are associated with a poor prognosis [23].

In addition to organ impairment caused by cGVHD, patients often have comorbidities associated with treatment, such as osteoporosis induced by corticosteroids [7]. Overall, cGVHD is associated with considerable patient burden and impacts health-related quality of life, depending on the clinical features experienced, and reduced health-related quality of life correlates with increased disease severity and lack of response to treatment [24, 25]. Patients frequently report reduced functional capabilities [26], psychological distress [27], and negative mood [26]. Furthermore, cGVHD has been associated with diagnoses of disabilities including keratoconjunctivitis sicca, sclerosis, and reduced lung function [28, 29]. Uninsured patients may also face a substantial financial burden from cGVHD due to high treatment costs [30].

Standard GVHD prophylaxis for HLA-matched transplants comprises a calcineurin inhibitor plus a short course of methotrexate or mycophenolate mofetil (MMF) with or without additional antithymocyte globulin (ATG) in transplants from unrelated donors; ATG has recently been also recommended for use in HLA-identical sibling transplantation [31]. The aim of these prophylactic treatments is to reduce the risk of aGVHD, one of the major risk factors for cGVHD which continue to manifest in approximately half of the patients [32]. Nevertheless, several approaches have resulted in decreased rates of cGVHD, including the use of ATG as part of GVHD prophylaxis [33–36] and post-transplant cyclophosphamide in combination with a calcineurin inhibitor (with or without MMF), even in the peripheral blood stem cell transplantation setting and naïve T-depleted grafts [37]. Post-transplant cyclophosphamide has also increased the number of haplo-identical donors without any increases in rates of cGVHD [38, 39]. However, a significant percentage of patients will develop moderate-to-severe cGVHD [40, 41]. Early intervention can ameliorate symptoms and improve survival rates for patients who develop cGVHD [5]. Patients with mild cGVHD may only require treatment with local therapies such as topical steroids, depending on the organ or site affected and on the risk of relapse of the underlying disease [42]. The recommended first-line treatment for moderate or severe disease is systemic corticosteroids (prednisone) with or without a calcineurin inhibitor [3, 31, 42]. Overall, only 40–50% of patients respond adequately to first-line treatment, and over half become steroid-resistant or -dependent, requiring second-line treatment within 2 years due to suboptimal responses, loss of response, or unsuccessful steroid tapering [3, 6]. Additionally, the long-term use of immunosuppressants, including steroids, has been associated with significant toxicity and increased risk of infection [10].

The mechanisms of steroid resistance in cGVHD are not well described [43], and prognosis remains poor for individuals with SR-cGVHD. Steroid resistance has been characterized in many inflammatory diseases such as asthma, chronic obstructive pulmonary disease, and rheumatoid arthritis [44]. To date, several underlying mechanisms have been identified for these conditions, including activation of mitogen-activated protein kinase, reduced histone deacetylase-2 expression, activation of transcription factor activator protein 1, and increased P-glycoprotein-mediated drug efflux [44]. It is yet to be investigated whether any of these mechanisms play a role in GVHD. While we are not aware of any studies into the mechanisms of steroid resistance in cGVHD, murine models have been developed for aGVHD [43]. Despite the key role of T cell responses in aGVHD, these models showed no significant association with donor T cell characteristics, inflammatory cytokine levels, or timing of steroid initiation [43], whereas increasing evidence underlines the role of myeloid cells and fibroblasts [45]. While steroid-resistant cGVHD, as defined by a lack of response to steroids, is challenging, a significant number of patients respond to a regimen of increased steroids. However, exacerbations of cGVHD are frequent when steroid withdrawal is attempted, resulting in significant long-term morbidity due to prolonged use of steroids. Research on the mechanisms of steroid-refractoriness and -dependence remains ongoing and will aid in developing treatments to overcome steroid resistance. Another clinical challenge in SR-cGVHD remains the non-reversibility of certain organ manifestations [24, 46], such as severe ocular involvement which rarely responds to immunosuppressive treatment, and deep cutaneous sclerosis showing a protracted response, if any [3]. The same applies for pulmonary manifestations, with stabilization of the condition regarded as a success, which impairs appraisal of the treatment efficacy [47].

There are no standard second-line treatments for patients with SR-cGVHD. While an increasing number of treatment options are becoming available, data are limited, and no consensus has been found on an optimal approach; these circumstances lead to a wide variation in individual clinical practice [3, 31, 48]. Numerous clinical trials have been performed to evaluate interventions [49], and results from retrospective and prospective studies often report high response rates, but are difficult to interpret due to variations in study design and populations treated [50]. Table 1 outlines some of the main second- and third-line treatment options currently available for SR-cGVHD. The choice of treatment for SR-cGVHD is largely patient-specific and based on several factors, including clinical experience and published evidence, risk profile, disease history, comorbidities, individual tolerance to medication, and access to ongoing clinical trials [51]. Currently, ibrutinib, a Bruton’s tyrosine kinase and interleukin-2-inducible T cell kinase inhibitor, is the only FDA-approved therapy for SR-cGVHD. Ibrutinib targets both B and T cells, making it an attractive option for manifestations that involve auto-antibody production.

**Table 1:** Therapy options for steroid-refractory cGVHD.

Therapy	Type	Recommendation	Evidence	Overall response^a^	Overall survival^a^	Toxicities	Study type
Ibrutinib	Bruton’s tyrosine kinase inhibitor	2nd line	III-1	BOR 67% (CR 21%, PR 45%) in 42 patients with cGVHD with median follow-up of 13.9 months [63]	71% at 2 years in cGVHD [64]	Pneumonia, impaired platelet function [51]	Phase 2a trial
Extracorporeal photopheresis	UVA treatment of mononucleated blood cells via leukapheresis	2nd line	II	Rates dependent on site and severity— highest responses in skin, liver, mouth, and BOS [56, 58, 71, 72]; 67% (CR 23%, PR 44%) in 48 patients with SR-cGVHD [71]	53–78% at 1 year [3, 56]	Vascular access complications [51]	Phase 2 randomized trial
Mycophenolate mofetil	Antimetabolite immunosuppressant	2nd line	III-1	26–64% [3]	67–96% at 1 year [3]	Viral reactivation, hypertension, pneumonia, post-transplantation lymphoproliferative disease [51]	Retrospective cohorts
Rituximab	CD20 (B cell surface antigen) monoclonal antibody	2nd line	II	65% in 38 patients with SR-cGVHD [73]; 70% (CR 10%) in 20 patients with SR-cGVHD [67]; 27% in 37 patients with sclerotic cGVHD [68]; 17% (CR 17%) in 6 patients with SR-cGVHD [68]	72% at 1 year; 76% at 2 years [3, 73]	Infections, infusion-related symptoms, late neutropenia [67, 72]	Phase 2b randomized trial
Ruxolitinib	Janus kinase 1/2 inhibitor	2nd line	II	BOR 76% (CR 12%, PR 64%) in 165 patients SR-cGVHD [52]; 85% (CR 7%, PR 78%) in 41 patients with SR-cGVHD [73]	97% at 6 months [73]	Viral reactivation/infection, peripheral neuropathy, anemia, thrombocytopenia, and neutropenia [52, 74, 75]; viral reactivation, cytopenia, malignancy relapse [73]	Phase 3 randomized trial
mTOR inhibitors (sirolimus, everolimus)	mTOR inhibitor	2nd line	III-1	81% (CR 38%, PR 43%) in 47 patients with SR-cGVHD [76]; 94% of 16 patients with cGVHD [77]	–	Thrombotic microangiopathy, renal insufficiency, proteinuria [76–78]	Phase 2a trials
Imatinib	Multikinase inhibitor	2nd line	II	79% (CR 37%, PR 42%) in 19 patients with SR-cGVHD [79]; 26% in 35 patients with sclerotic cGVHD [68]	84% at 1.5 years [79]	Fluid retention, myelosuppression, anemia [79]	Phase IIb trial
Methotrexate	Antimetabolite	2nd line	III-1	83% (CR 62%, PR 21%) in 86 patients [80]	96% at 1 year, 90% at 1.5 years [80]	Hepatotoxicity, leukopenia, thrombocytopenia [81, 82]	Retrospective cohorts
Pentostatin	Adenosine deaminase inhibitor	>2nd line	II	55% in 58 patients with SR-cGVHD [83]	78% at 1 year; 70% at 2 years [83]	Infections [83]	Phase 2a trials
IL-2 therapy	IL	> 2nd line	III-1	52% [3]	Under investigation in phase 1 and 2 clinical trials [3]	Injection site induration, infections [51]	Phase 2 trial
Pomalidomide	Glutamic acid derivative, TNF-α inhibitor	>2nd line	III-1	7 of 13 patients had PR [84]; ORR 47% (PR 100%) in 32 patients with cGVHD [85]	In a phase 1/2 study, all responders were still alive after a median follow-up of 4.6 years [84]	Lymphopenia, neutropenia; thalidomide toxicities not observed; adverse events included infections, muscle cramps, and fatigue; early use after transplant may increase risk for inflammatory flares [84, 85]	Phase 2 trial
Ixazomib	Proteosome inhibitor	>2nd line	III-2	40% of 50 patients had PR/CR [86]	90% at 12 months [86]	–	Phase 2 trial
Low-dose total lymphoid irradiation	Radiation therapy	>2nd line	III-2	54% of 13 patients with cGVHD achieved PR [87]; 75% of 12 patients achieved clinical response at 6 months [88]	Median 13 months (range, 3–113 months) in responders vs 10 months (range, 0–41 months) in non-responders [87]	Thrombocytopenia, neutropenia [87, 88]	Retrospective cohorts
Mesenchymal stem cells	Stem cells	>3rd line	III-2	74% (CR 21%, PR 53%) in 19 patients with SR-cGVHD [89]	78% at 2 years [89]	None reported [89]	Phase 2 trial
Thalidomide	Glutamic acid derivative, TNF-α inhibitor	>3rd line	II	38% (CR 3%, PR 35%) of 37 patients with SR-cGVHD [90]	41% at 2 years in SR-cGVHD [90]	Birth defects, constipation, rash, fatigue, somnolence, and neuropathy	Phase 2 trial
Alefacept	T cell activation inhibitor	>3rd line	III-2	8 of 12 patients showed a response, with 2.25 weeks as median time to response [91]	50% at 30 months [91]	No dose-limiting toxicities [91]	Phase 1 trial
Abatacept	T cell activation inhibitor	>3rd line	III-2	44% of 16 patients with SR-cGVHD achieved PR [92]	–	No dose-limiting toxicities were identified [92]	Phase 1 trial
Tocilizumab	Anti-IL-6 receptor antibody	>3rd line	III-2	70% of 11 patients achieved PR [93]	82% with median follow-up of 22 months [93]	Infections, granulocytopenia, thrombocytopenia [93]	Retrospective cohorts
Cyclophosphamide (either pulse of low dose)	Alkylating agent	>3rd line	III-2	100% of 3 patients with cGVHD showed response in treatment of skin and oral cavity [94]; 60% of 15 patients showed improvement after 8–12 monthly cycles [95]	–	Short-term myelosuppression, neutropenia, fatigue, nausea [94–96]	Retrospective cohorts
Baricitinib	Janus kinase 1/2 inhibitor	>3rd line	III-2	90% of 20 patients with SR-cGVHD at any time during the study [97]	FFS 74% at 1 year, 37% at 2 years [97]	Viral reactivation, neutropenia, hypophosphatemia, hypertriglyceridemia, upper respiratory tract infections [97]	Phase 1/2 single arm clinical trial
Belumosudil	ROCK2 inhibitor	Available in clinical trials only	III-1	74% (CR 3%, PR 71%) of 132 patients with cGVHD [98]	FFS 77% at 6 months [98]	Pneumonia, hypertension, hyperglycemia, increased gamma-glutamyltransferase [98]	Phase 2 open-label, randomized clinical trial
Axatilimab	IgG4 antibody targeting the CSF-1 receptor	Available in clinical trials only	III-2	58% of 12 patients with cGVHD across doses [99]	–	Increased gamma-glutamyltransferase, asparatate aminotransferase, and creating phosphokinase, periorbital edema [99]	Phase 1/2 dose-escalation and dose-expansion study

*BOR* best overall response, *BOS* bronchiolitis obliterans syndrome, *CD* cluster of differentiation, *cGVHD* chronic graft-versus-host disease, *CR* complete response, *CSF-1* colony-stimulating factor 1, *FFS* failure-free survival, *Ig* immunoglobulin, *IL* interleukin, *mTOR* mammalian target of rapamycin, *ORR* overall response rate, *PR* partial response, *ROCK2* rho-associated coiled-coil kinase 2, *SR-cGVHD* steroid-refractory chronic graft-versus-host disease, *TNF-α* tumor necrosis factor alpha, *UVA* ultraviolet A.

II: evidence from more than one well-planned non-randomized clinical trial, from cohort or case-controlled analytic studies (preferably at several sites); III-1: several reports from retrospective evaluations or small uncontrolled clinical trials; III-2: only one report from a small uncontrolled clinical trial or retrospective evaluations [49].

^a^Values are given for cGVHD patients (steroid-refractory and steroid-dependent) unless otherwise stated.

In the second section of this review, we discuss three case studies of patients with SR-cGVHD from our clinics and suggest potential treatment options based on their individual profiles.

## Case presentation

### Patient 1

Patient 1 is a 74-year-old male former smoker who received a peripheral blood HLA-mismatched graft from an unrelated female donor. He was initially treated for quiescent onset of moderate cGVHD (skin grade 2, mouth grade 1, and lung grade 1) starting 10 months after transplantation, with 1 mg/kg/day prednisone tapered to 0.25 mg/kg/day plus tacrolimus and fluticasone, azithromycin, and montelukast therapy. He then developed a > 15% decline in forced expiratory volume in 1 second (FEV_1_) with steroid taper, FEV_1_ of 44%, FEV_1_/forced vital capacity of 0.62 (grade 2), moderate mouth symptoms with lichenoid features and ulceration (grade 2), and deep sclerotic features on lower limbs (grade 3). He has an active fungal lung infection and confirmed cytomegalovirus reactivation.

### Treatment options

Patients with SR-cGVHD should ideally be enrolled in a clinical trial; however, this case would most likely not meet eligibility criteria due to the active fungal infection. Despite the greater efficacy of ruxolitinib than best available therapy in patients with SR-cGVHD [52], the patient’s active fungal and viral infections cause some reluctance for use of ruxolitinib, ibrutinib, or MMF due to a further increase in risk for exacerbation of the ongoing infections. Given the severity of his baseline airway obstruction, the possibility of further bronchiolitis obliterans syndrome progression despite active fungal infection, and his poorly controlled cGVHD (affecting extrapulmonary sites), we would favor prompt initiation of extracorporeal photopheresis (ECP). ECP has been assessed in both steroid-dependent- and SR-cGVHD patients [53] and has demonstrated efficacy and safety in the treatment of bronchiolitis obliterans syndrome following HSCT and lung transplantation [54, 55]. It has also shown significant response rates in patients with sclerodermatous and mucosal GVHD in previous studies, including a randomized phase 2 trial [53, 56]. ECP increases regulatory T cells, with beneficial effects reported in patients with sclerotic SR-cGVHD [53]. ECP is associated with very low rates of infectious complications and has a steroid-sparing effect in cGVHD, which is particularly appropriate for this case [53, 55]. Among the many different schedules reported for ECP delivery, we usually administer it twice weekly for the first month and then twice weekly every other week for 6 to 12 months. Once ECP is initiated, we also pursue steroid tapering as soon as possible, to reduce the impact on the treatment of his fungal infection and to avoid new cytomegalovirus reactivation episodes. To monitor lung response and guide steroid tapering or further therapy, we would order pulmonary function tests at least every 4 weeks during the first 3 months. We would consider ECP discontinuation only if GVHD progresses after at least 3 months of therapy.

### Patient 2

Patient 2 is a 58-year-old male who received a reduced-intensity peripheral HSCT graft from his HLA-identical sister and GVHD prophylaxis with cyclosporine (CSP) and MMF. He developed steroid-sensitive classic grade 2 aGVHD (skin grade 2, gut grade 1) on day + 53 and presented 18 months after transplantation with quiescent onset of high risk cGVHD, including elevated bilirubin > 3 mg/dl (grade 3) plus fasciitis affecting wrists, elbows, and shoulders, with moderate limitation in the photographic range of motion scale (P-ROM) of 16 (grade 2), while still receiving treatment with low-dose corticosteroids and sirolimus. Both episodes (aGVHD and cGVHD), which were initially treated with prednisone 1 mg/kg/day, were complicated by latent steroid-psychosis requiring a rapid taper of steroids and combination treatment. The patient also experienced impaired renal function on CSP. At the time of cGVHD progression, immunoglobulins (Ig) were above the normal range, with concurrent elevated IgG1 and IgG2 deficiency. The patient also had an expanded CD19+ B cell count, normal CD4+ T cell counts, platelets 75/nl, and granulocytes 1.5/nl. Screening for liver-directed autoantibodies confirmed the presence of a significant titer of antinuclear and anti-smooth-muscle antibodies.

### Treatment options

In this case, as the patient is thrombocytopenic, FDA-approved ibrutinib is not the most suitable option. While ibrutinib targets both B cells and plasmablasts, it also interferes with platelet function, which may increase the risk of bleeding complications [57]. While MMF could be an option because of evidence from autoimmune hepatitis [58], the potential cytostatic effect of MMF may promote cytopenia already present. Additionally, while ECP could be beneficial for this patient, it requires time to reach a response and may be ineffective when given alone.

Therefore, we decided to treat the patient with a course of rituximab combined with ruxolitinib and to continue low-dose steroids because of prior intolerance. While ruxolitinib has been shown to be an effective immunosuppressive agent targeting T cells and macrophages (both involved in cGVHD, including sclerosis), it also may indirectly target B cells, which are thought to be involved in this patient, by blocking follicular T helper cells inducing new B cells [59]. The combination would permit the initial depletion of a significant proportion of autoreactive B cells followed by a blockage of new “supply”. Moreover, ruxolitinib has been associated with reactivation of prior seroconverted hepatitis B, indicating a suppressive effect on the humoral-mediated immune mechanism [60]. However, this strategy poses significant infectious risks, as both agents have been associated with infectious complications [61]. Therefore, proper infection prophylaxis (*Pneumocystis jirovecii* pneumonia, varicella-zoster virus) and continuing antibiotic prophylaxis is mandatory, and substitution of Ig in the presence of bacterial infection complications to resolve the IgG2 deficiency is indicated. Ruxolitinib also requires monitoring of blood counts and liver enzymes, as the patient already has cytopenia and impaired liver function, predisposing him to side effects.

### Patient 3

Patient 3 is a 66-year-old female who received a matched unrelated donor peripheral blood HSCT. She has active cGVHD with fibrotic and inflammatory manifestations despite 3 months of treatment with prednisone 0.5 mg/kg/day and continued prophylactic CSP. Five months post-transplant, she presented with the following symptoms: morphea-like superficial sclerotic features in the skin plus lichen sclerosus (40% body surface; grade 2); moderate mouth symptoms with lichenoid features (grade 2); vulvar lichen sclerosus-like features and mild discomfort (grade 1); and moderate dry eye symptoms with corneal keratinization but no vision impairment (grade 2). Pre-existing comorbidities include type 2 diabetes, avascular necrosis, and neutropenia.

### Treatment options

Due to lack of evidence to guide which therapy is superior for this patient specifically, we would first consider treatment in a clinical trial as an option for this case. We would replace CSP with another treatment, as CSP failed to control the progression and potentially impedes the emergence of regulatory T cells, which are involved in the pathobiology of cGVHD [24]. Another consideration is to add a secondary therapy with a steroid-sparing effect, as this patient has diabetes and is at increased risk for prolonged systemic treatment including corticosteroids, because she received a peripheral blood HSCT instead of bone marrow transplant [62]. Finally, the treatment for this patient should be an agent that targets the pathobiology of sclerotic cGVHD phenotypes and has a reported beneficial effect in this clinical setting.

Several treatment options are available for this case. Ruxolitinib targets several signaling pathways involved in the pathobiology of cGVHD and has demonstrated superior efficacy than the best available therapy in patients with cGVHD in a recent phase 3 clinical trial [52]. Ruxolitinib may therefore be the preferred therapy option for this case, although the patient should be monitored for neutropenia and increased infection risk [52]. Alternatively, ibrutinib targets T and B cell signaling pathways and has reported responses in patients with sclerotic manifestations [63, 64]. We would start ibrutinib at a dose of 280 mg once daily plus anti-mold infection prophylaxis (i.e., voriconazole 200 mg twice daily, or posaconazole 100 mg twice daily), because of the concern of early invasive fungal infections [65]. Other treatment options include ECP or other treatments that increase regulatory T cells, such as interleukin-2, which has also shown benefit in patients with sclerotic SR-cGVHD [66]. Rituximab, which has demonstrated initial high responses in retrospective studies (but lower responses in prospective studies) for sclerotic SR-cGVHD [67, 68] could also be considered if the treatments previously discussed are not suitable. Finally, MMF in combination with steroids could also be an option if other options are not feasible, but its efficacy for sclerotic cGVHD has not been tested prospectively.

A final important point for management of this case is to optimize supportive care [69], such as through the use of topical dexamethasone oral rinses together with oral nystatin to prevent/treat superimposed yeast infection for oral manifestations, application of topical betamethasone for the vulva [69], and supportive ocular treatment including the use of eye lubricant, CSP ophthalmic emulsion, ocular punctal plugs, scleral lens, and other care [70].

## Conclusion

In summary, these three patient cases illustrate multiple options available for patients with SR-cGVHD. While there is one FDA-approved treatment currently available, enrollment in ongoing clinical trials is also an important option for eligible patients whose treatment has failed on one or more previous therapies. In the absence of robust evidence of benefits for any one intervention, treatment choices should be based on physician experience, ease of use, need for monitoring, risk of toxicity, and potential worsening of pre-existing comorbidities.
